# Economic Analysis as a Basis for Large-Scale Nitrogen Control Decisions: Reducing Nitrogen Loads to the Gulf of Mexico

**DOI:** 10.1100/tsw.2001.270

**Published:** 2001-10-23

**Authors:** Otto C. Doering, Marc Ribaudo, Fransisco Diaz-Hermelo, Ralph Heimlich, Fred Hitzhusen, Crystal Howard, Richard Kazmierczak, John Lee, Larry Libby, Walter Milon, Mark Peters, Anthony Prato

**Affiliations:** Agricultural Economics Department, Purdue University, West Lafayette, IN 47906, USA

**Keywords:** nitrogen, hypoxia, Mississippi Basin, economics, nutrient management, wetlands, agriculture

## Abstract

Economic analysis can be a guide to determining the level of actions taken to reduce nitrogen (N) losses and reduce environmental risk in a cost-effective manner while also allowing consideration of relative costs of controls to various groups. The biophysical science of N control, especially from nonpoint sources such as agriculture, is not certain. Widespread precise data do not exist for a river basin (or often even for a watershed) that couples management practices and other actions to reduce nonpoint N losses with specific delivery from the basin. The causal relationships are clouded by other factors influencing N flows, such as weather, temperature, and soil characteristics. Even when the science is certain, economic analysis has its own sets of uncertainties and simplifying economic assumptions. The economic analysis of the National Hypoxia Assessment provides an example of economic analysis based on less than complete scientific information that can still provide guidance to policy makers about the economic consequences of alternative approaches. One critical value to policy makers comes from bounding the economic magnitude of the consequences of alternative actions. Another value is the identification of impacts outside the sphere of initial concerns. Such analysis can successfully assess relative impacts of different degrees of control of N losses within the basin as well as outside the basin. It can demonstrate the extent to which costs of control of any one action increase with the intensity of application of control.

## INTRODUCTION

Economics is unlikely to be used as the basis for large-scale nitrogen (N) control decisions. The first level of decision criteria is likely to be determined by hydrologists, engineers, and others based on what is technologically possible, and then public perception is the ultimate determining factor. The critical contribution economics can play is by identifying the relatively cost-effective opportunities to achieve a public goal among the technically viable alternatives. The economic analysis can bound some of the choices available, but it is very unlikely to determine them. The choices will be made by decision makers based on public perceptions and values. The case in point is hypoxia in the Gulf of Mexico. Critical aspects of the science are not certain and are part of a continuing controversy[1,2,3]. The purpose of this article is to review that example and illustrate the relationship between the scientific information, the uncertainties surrounding some important parameters, and how economics can still be useful given the uncertainties related to N. The debate in *Science* over the linkage between hypoxia and N flows to the Gulf is unlikely to be resolved, at least in the near term. Part of that debate relates to assessing causality or blame[3]. Our contention is that economics can still be useful in the dilemma of uncertain science in guiding some steps that work toward solutions (irrespective of blame).

A so-called Dead Zone has become a dominant feature of the northern Gulf of Mexico, attributed largely to N loads from the Mississippi River[4]. The northern Gulf of Mexico's zone of oxygen (O)-deficient water represents one of the largest hypoxic zones in the western Atlantic Ocean[5]. At its peak, this zone stretches along the inner continental shelf from the Mississippi Delta westward to the upper Texas coast, covering about 7000 $mi^2$. The hypoxic zone is caused by the interaction of several features of the northern Gulf. During the summer months, the waters in the Gulf are warm and relatively stable. During this time, freshwater inflows from the Mississippi River, which are lighter than salt water, form a layer at the surface that is rich in inorganic N carried down the river. The warm waters and availability of nutrients greatly increase the primary productivity (eutrophication) of the upper waters. Phytoplankton and organic carbon from zooplankton sink to the bottom and utilize O through respiration and decay. Without adequate mixing with the upper waters, dissolved O near the bottom decreases to hypoxic or anoxic levels.

One critical determination is the identification of N sources. This has been controversial in terms of the Gulf's hypoxia from the standpoint of both attribution of sources and past perceptions that were at odds with current data[3]. The relative magnitude of different sources is also important. Are we dealing with an elephant or a breadbasket? From a cost and technical efficiency perspective, it often pays to go after the big or concentrated sources. The economics of pollution control are such that it is typically less expensive to reduce a given proportion of a high concentration as compared with a low concentration. In addition, the cost of reducing a pollutant often increases rapidly as the concentration decreases.

Integrated assessments of large-scale environments or large pollution flows can sometimes be less than precise in their determination and still provide the critical information policy makers need to make decisions. The level of precision needed for decision making is also related to the public perception of a problem and the level of public determination to do something about it. In some cases, demonstrating positive benefit/cost results from public action may not be important to the public if the problem is perceived to be important enough. The public determination may be that it should be fixed, and some action should be undertaken to set that in motion irrespective of normal cost considerations. The national hypoxia assessment process provides an excellent example of both the limited role of economic analysis in the public perception about the decision that something must be done and the fact that economics does have something to say in how the problem might be tackled even though some of the science is controversial and uncertain.

## DETERMINATION OF SOURCES AND SOURCE CHARACTERISTICS

Determination of the N sources is a critical first step. The normal expectation is causality between source and problem. This is the argument in *Science* that may never be completely settled, but need not necessarily be settled for public action to occur. Public action does not require or often achieve 100% certainty as its base. However, if one is to determine the costs of reducing N losses, source determination and source characteristics become important parts of that exercise. Geography, timing, and intensity are also critical. In the identification of sources, it is not only the source and relative magnitude of N inputs into the basin that are important but also the relative magnitudes of sources of the inputs that ultimately enter the Gulf of Mexico[6]. These are different in some important ways because of transport and other factors.

Table 1 gives the N mass balance data for the river system, as estimated by Goolsby et al.[6]. It is clear from the table that fertilizer (6495 thousand tonnes/year), the soil system (6464 thousand tonnes/year), legumes (4327 thousand tonnes/year), and animal waste (1296 thousand tonnes/year) are major sources of N in the Mississippi drainage basin. However, only a portion of the total N inputs is discharged into the Gulf via the Mississippi-Atchafalaya River (1567 thousand tonnes/year) and atmospheric deposition (15 thousand tonnes/year).

In Table 2, a summary of the N input sources is compared to the results of an analysis of the contributions of sources to the total N yield to the Gulf conducted by Goolsby et al.[6] The distinctions made here become critical in assessing where N losses to the Gulf are best targeted as compared to inputs into the basin. The fertilizer-soil system (50%) still represents the 800-pound gorilla with respect to concerns about N. As a source of N inputs to the Gulf, the fertilizer-soil system is a slightly less important contributor than it is in providing N inputs to the basin. Basing policy on inputs to the basin rather than loadings to the Gulf would still result in the same policy target.

Table 2 shows that animal manure is more of a factor in terms of its N contribution to the Gulf than it is an input to the basin. Location may be the factor that brings this about. Animal manure also illustrates the complexity of determining sources and their relative priority for actions to reduce their N losses. Historically, the amount of animal manure N contributed to the basin has been roughly the same over the last 50 years[6]. With this in mind, one might assume its importance to N delivered to

**TABLE 1**
**Nitrogen Mass Balance Data for Mississippi-Atchafalaya River Basin for 1980–96, Except as Noted**

| | |
|---|---|
| **New N inputs (1000 metric tons/year)** | |
| Fertilizer | 6,495 |
| Total fixed from legumes | 4,327 |
| Atmospheric deposition (1990–1996 average) (includes wet + dry nitrate) and organic N | 1,411 |
| **Recycled N inputs** | |
| Manure - (total adjusted for volatilization losses) | 1,296 |
| Potentially mineralizable from soil | 6,464 |
| Atmospheric deposition – wet ammonia | 651 |
| Point source inputs to streams | 287 |
| Total annual new and recycled inputs | 20,931 |
| **Annual outputs (1000 metric tons/year)** | |
| Atmospheric deposition on Gulf | 15 |
| Mississippi-Atchafalaya River discharge (1980–1996 average) | 1,567 |
| Manure volatilization loss | 1,488 |
| Fertilizer volatilization loss | 133 |
| Total crop and pasture removal | 9,658 |
| Plant senescence | 3,326 |
| Denitrification from cropland soil | 1,704 |
| Immobilization in soil organic matter | 2,978 |
| Total outputs | 20,869 |
| **Residual (inputs – outputs) difference** (@ 0.3%) | 62 |

From Goolsby et al.[6].

**TABLE 2**
**Sources of Nitrogen Contributed to the Mississippi River Basin and Gulf of Mexico**

| N Source as a Percent of Total: | Inputs to Gulf | Inputs to Basin |
|---|---|---|
| Fertilizer-soil system | 50% | 61.9% |
| Animal manure | 15% | 6.2% |
| Municipal/industrial point sources | 11% | 1.4% |
| Legumes | Not significant | 20.7% |
| Atmospheric deposition and unmeasured inputs such as ground water | 24% | 9.9% |

From Goolsby et al.[6].

the Gulf was constant. However, the hog industry, a major manure producer in the basin, has been undergoing rapid structural transformation. Thus structural change affects the potential impact of thus industry's manure on the Gulf.

The key question becomes whether continued structural concentration in the industry will increase (or decrease) the amount of N delivered to the Gulf because of location or other factors, even if the total volume of manure in the basin remains the same.

Concern about future delivery of N from manure to the Gulf might require more stringent location and management controls under the new structure of the industry than would have been required under the more traditional structure. So, not only might animal manure contribute a higher proportion of the N delivered to the Gulf than it inputs to the basin, but changes in the regulation and management of the industry have the potential to increase that amount further (or reduce it).

Municipal and industrial point sources were a point of controversy during the assessment process. The perception was and is that municipal sewage and some industrial sources are major inputs to N in the Gulf. The Goolsby balance sheet indicates that these sources contribute between 1 and 2% to the total N that enters the basin[6]. The perception of these point sources as important contributors is more credible in the context of the 11% of the total that these sources contribute as N inputs to the Gulf as indicated in Table 2. What is key here is that the municipal and industrial sources, because of location and character, contribute their flow of N directly to the Gulf with little amelioration due to transport. The same arguments and controversy surround atmospheric deposition and some other sources. Again, these are much more important as inputs to the Gulf than as inputs to the basin. In contrast, while N from legumes is an important input into the basin, it cannot be identified as a separate significant input into the Gulf, as it is likely bound in soil systems.

Location is critical not only for its impact on the importance of different sources but also for its importance in targeting control strategies. Goolsby et al.[6] show the geographical concentration of N losses within the basin in the Upper Midwest. Much of this is due to high fertilizer use, the nature of the soil system, and animal production, but it also includes the large contribution of the Chicago River from Chicago's sewage. If one is to spend resources attempting to reduce N losses, these may be best spent where loadings are highest.

External influences like weather and response time lags are additional critical and confounding factors with respect to the impacts of different sources. Fig. 1 illustrates the importance of stream flow on N flux. In essence, the amount of N delivered to the Gulf is also determined by stream flow as a result of changing rainfall levels. In addition, we have a critical time factor in terms of the buffering activity of the soil system. A soil system may well leak continuing and relatively constant amounts of N for some years after additions of N are reduced or even eliminated. Fig. 2 shows the increase over time of N inputs into the basin, largely composed of additional N fertilizer. It also illustrates the buildup of residual N in the soil system when inputs of fertilizer N outpaced removals in the 1950s and 1960s. From the 1970s onward, as efficiency of N fertilizer use increased, weather and changes in crop yield dominated the increasing fluctuations in residuals.

The time delay and the dependence upon weather make both economic and policy calculations much more complex[7]. What we have is not a simple mechanistic relationship in which reductions in N losses in the basin are direct and proportional to our actions and the reduction to the N inputs to the Gulf is also correspondingly direct and proportional.

## ALTERNATIVE MEASURES TO REDUCE NITROGEN IN THE GULF

The research on alternative policies for reducing N loads to the Gulf of Mexico was conducted for the Gulf of Mexico Hypoxia Assessment managed by the White House Committee on Environment and Natural Resources starting in 1998. The effort included a series of six interrelated reports examining different aspects of the hypoxia issue. The economics team evaluated the social and economic costs and benefits of methods for reducing nutrient loads to the waters of the basin.

Two basic approaches were taken with respect to N flows into the Gulf in the hypoxia assessment[8]. One was to reduce the amount of N reaching the surface waters in the Mississippi Basin, the other was to reduce the N concentration already in the

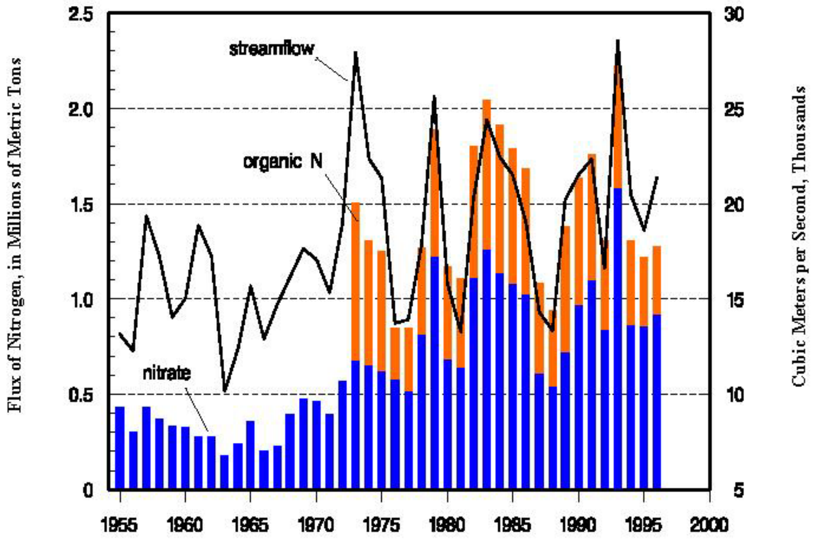

**FIGURE 1**. Annual flux of nitrate and organic N and mean annual stream flow from the Mississippi River Basin to the Gulf of Mexico. From Goolsby et al.[6].

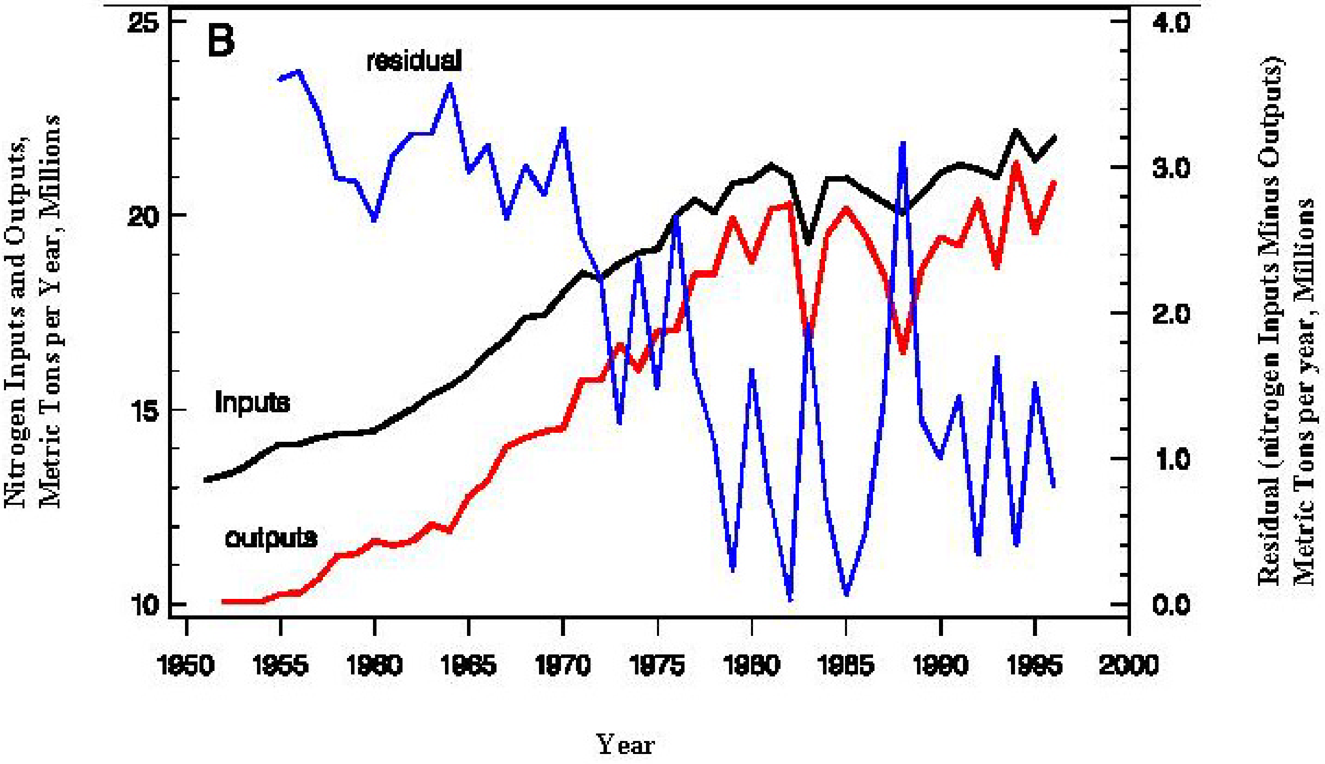

**FIGURE 2**. Annual N inputs and outputs for the Mississippi-Atchafalaya River Basic from all major sources. From Goolsby et al.[6].

waters within the basin flowing to the Gulf. Much of the effort on N load reduction was also focused on N from agriculture. The information presented in Table 2 on the inputs to the basin reflects priorities in N reduction. The recommended approaches that would yield the greatest potential reduction in N losses to the basin were N management on the farm (900,000 to 1,400,000 metric tons/year), substituting perennial crops for 10% of the present corn-soybean area (500,000 metric tons/year), and improving management of animal manure (500,000 metric tons/year)[8]. Creating and restoring 5 to 13 million acres of wetlands in the basin had a potential for N reduction of 300,000 to 800,000 metric tons/year with a similar reduction possible from the restoration of 19 to 48 million acres of riparian bottomland hardwood forest. The latter two approaches are interception strategies. Reducing point source N pollution was estimated to have a potential reduction of 20,000 metric tons/year[6]. The task of the economic assessment was to assess the cost effectiveness of these suggested strategies.

Among the specific recommendations for reducing N losses were changes in the design and operation of Mississippi River diversions in the Mississippi Delta, restoration of flood-prone lands in the Upper Mississippi, and tertiary treatment for new wastewater treatment plants in the basin[8]. Those agricultural management practices yielding the greatest potential for reducing N losses were a 20% reduction in fertilizer N application in the basin combined with other agricultural practices, such as optimum timing of application, alternative crops, wider spacing of tile drains, and better management of livestock manure. The 20% reduction in N fertilizer application was to be accompanied by proper crediting of N for legumes and manure and optimum timing of fertilizer application. The second measure having major impact would be the restoration or creation of 24 million acres (9.7 million ha) of riparian zones and wetlands to reduce N already in the surface water[8]. It would be important to strategically place these in watersheds for most effective N removal. One important judgment associated with these recommendations was the conclusion by the assessment team agronomists that N loss reductions from agriculture in the 20% range could be achieved with relatively small yield reduction[8]. This magnitude of reduction proved to correlate well with the economic impacts of N loss reductions.

## DESIGNING THE ECONOMIC ASSESSMENT

The approach of the economic assessment honored the causality implied by the N flow and hydrological assessments and focused on the economics of the methods suggested for reducing N loads to the waters in the basin. Key to the economic assessment was the incidence of costs or benefits in actions taken to reduce N losses[9]. An overall benefit/cost analysis of N reduction was not performed because of the difficulty of calculating reliable costs of N damage to the Gulf of Mexico. These costs would in turn become the benefits of N reduction.[10]. Instead, the focus of the economic analysis became the identification and analysis of cost-effective measures to reduce N losses in the basin while also analyzing the indirect as well as the direct consequences of such actions. Cost-effective approaches make sense when the damages are uncertain[7,11,12]. A cost-effectiveness analysis is designed to provide information about the economic trade-offs associated with different types of actions. Cost-effectiveness analysis is appropriate when it is impractical or impossible to calculate the complete value of benefits (in this case, reduction of damage in the Gulf of Mexico or in stream or watershed benefits in the basin) provided by the alternative actions or policies[13]. A policy can be considered cost-effective (on the basis of life-cycle cost analysis of competing alternatives) if it is determined to have the lowest cost for a given amount of benefit—however benefit is defined.

To a large extent, the analysis was limited by the tools at hand. We had the capacity to analyze the production and economic impacts of fertilizer reductions and some changes in cropping practices and cropping systems for the Mississippi River Basin. We had a similar capacity to analyze the creation of wetlands. The tools to perform such analysis had been developed by the Economic Research Service, USDA, for the purpose of analyzing farm commodity and conservation programs.

Our analytic needs were met by the U.S. Agriculture Sector Mathematical Programming (USMP) regional agricultural model. The USMP model is a spatial and market equilibrium model designed for general-purpose economic and policy analysis of the U.S. agricultural sector[9]. The economic units that can be analyzed with USMP include products, inputs, geographic areas, and supply/demand markets. Within the modeling framework, it was possible to place restrictions on N losses due to fertilizer use. To represent the economic impacts of increased wetlands, crop acres were taken out of production. It was not possible to estimate how field-level reductions in N loss would contribute to reductions in N loadings directly into the Gulf.

Five different policy scenarios were evaluated:

- Restricting N fertilizer use by 20 and 45%;
- Reducing field N losses by 20, 30, 40, 50, and 60% at the least cost (by adjusting all inputs, including N fertilizer);
- Restoring 0.4, 2, 4, and 7.3 million ha of wetlands at the least cost for N filtration;
- Installing 10.9 million ha of streamside buffers; and
- Combining a 20% N fertilizer reduction with 2 million ha of wetland restoration.

The programming simulation analysis provided the following for different N loss constraints or other N loss reducing actions in agriculture:

- Changes in prices, exports, and net returns to producers
- Changes in crop acres and crop mix
- Changes in nutrient (N and phosphorous [P]) and sediment loss
- Changes in consumer and producer surplus

The wetlands analysis coupled with the programming simulation provided:

- Restoration and enrollment costs
- Changes in crop acres and crop mix
- Changes in crop prices, exports, and farm net cash returns
- Changes in nutrient (N and P) and sediment losses
- Changes in consumer and producer surplus

## DIRECTION AND MAGNITUDE OF RESULTS

As N use was restricted or N losses were controlled, production declined and agricultural prices increased. For example, a 20% fertilizer restriction resulted in a 6% increase in corn prices and a 2% increase in wheat prices. A 40% restriction resulted in a 28% increase in corn prices and a 13% increase in wheat prices. This is after allowing for adjustments in international trade: decreased exports and increased imports. Increasing constraints led to changed cropping systems and land taken out of production. Beyond a 40% N loss restriction, net returns to crops in the Corn Belt began to decline while the U.S. total continued to climb. Returns to livestock production declined steadily from the outset due to increased feed prices.

Similar patterns of response occurred with the expansion of wetlands to reduce N in surface waters. One of the dilemmas of wetland restoration/creation was targeting. From a technical efficiency standpoint, one would desire to concentrate wetlands in areas of high N concentrations in surface water. However, easement costs for wetlands are the major cost, and as one attempts to buy easements in a limited geographical area, one quickly absorbs the less valuable land and ends up bidding for higher priced land. This fact sets the technical goals against the economic ones and limits the extent to which targeted geographical concentration of wetlands is economically feasible.

One of the striking results was the divergence of impacts within the basin and outside the basin. Restriction to crop production within the basin (where the bulk of the major commodities is grown) increased prices and thus encouraged greater intensity of fertilizer use and increased production outside the basin where N losses increased. Increasing N restrictions also resulted in reduced P loss within the basin and increased loss outside the basin. However, since the bulk of the production is in the basin, net U.S. P losses decreased—as was the case with N losses and soil erosion as well. Increases in N and P loss outside the basin could result in water quality degradation there. This is an unintended consequence of a policy to reduce N in the basin that should be considered in considering alternative policy approaches.

Whatever major approach was taken to reduce N losses to the Gulf, costs increased at an increasing rate as any single approach was pushed toward its limit. As Fig. 3 illustrates, except for a very limited wetland program (in which the least expensive land was utilized), a program of reducing overall N losses on the farm was the most cost effective. Straightforward N fertilizer restrictions were not as cost effective because they limit the flexibility of producers. The logic of this is that programs for the former resulted in management attempting to optimize income while achieving a N loss reduction through a variety of measures like changing crop mix, tillage practices, and land use and using less fertilizer. For the last measure, the N fertilizer restriction, the management goal is to profit in spite of the direct

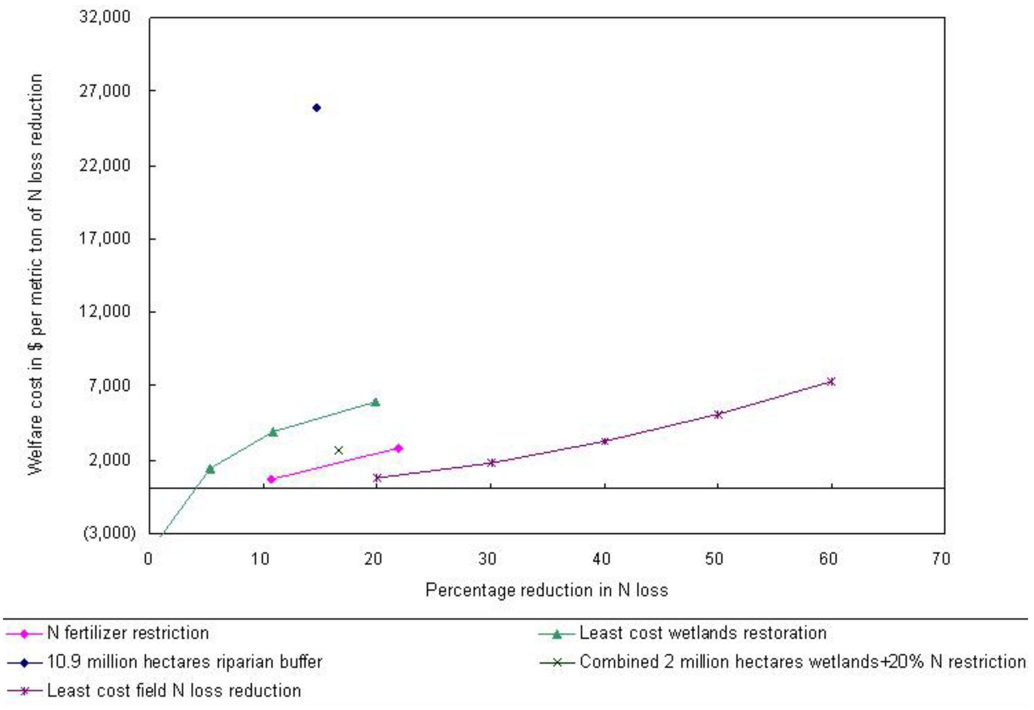

**FIGURE 3**. Agricultural sector adjustment costs per unit N reduction (net of intrabasin benefits, by control method). From Doering et al.[9].

N restriction, whether the resulting action reduces N losses or not.

A critical decision factor between the two agricultural N strategies would be getting either one to operate successfully. Few consider the cost of enforcing an absolute N restriction, and the cost can be high. On the other hand, an extensive and costly education and incentive program would be necessary to achieve high levels of participation in efforts to manage production to achieve given reductions in N losses.

The trade-off between restoring wetlands and reducing N losses at the source may not be as clear-cut as the cost comparison indicates. Limited wetland restoration (0.4 million ha) at low cost has negative overall costs because of the associated environmental benefits (such as enhanced wildlife habitat). So, some amount of wetland restoration makes sense. However, the land costs climb as one analyzes programs of 2, 4, and 7.3 million ha, respectively. This is an instance in which uncertainties in what is known about the effectiveness of different N loss reducing efforts, uncertainty in the benefits of each approach, and uncertainty about cost argue for a program that does some of each at a modest level.

## CONCLUSIONS AND RECOMMENDATIONS

The economic analysis did not yield results with a level of confidence that would have allowed calculation of optimal solutions, given the biophysical, cost, and benefit uncertainties. The analysis also demonstrated the complexity of direct and indirect impacts that would cast doubt on any attempt to identify a particular policy or goal as "optimal." However, the economic results did yield parameters within which cost-effective alternatives might be compared and a "superior" policy crafted. Some of these are:

- Marginal costs of N loss control increase as one measure or action is increasingly applied.
- Given what we understand and had to assume about the technical efficacy of different actions, a program goal of reducing N losses at the source appeared to be more cost effective.
- Very modest wetland reclamation was also cost effective, but with similar caveats.
- Economic impacts of N loss reductions on farmers and consumers and the shifts in out-of-basin behavior of agricultural producers are moderate with control up to the 20 to 30% N loss reduction level. For farmers, this level of control results in levels of price fluctuations and acreage and crop shifts within their previous experience driven by other factors over the preceding decade.

However, from what we know about the science, we cannot be certain that a given reduction in N losses would result in a given reduction of the hypoxic zone in the Gulf of Mexico. Uncertainty about the degree and timing of reductions in the hypoxic zone given improved management on cropland, uncertainty about the benefits of reduced hypoxia, sharply increasing marginal costs and impacts to the agriculture sector, and lack of knowledge about program costs suggest moderation is prudent.

The public may be concerned with environmental risk as well as with actual damage. It may be difficult to demonstrate to the public what damage there is today due to the current N

levels. Nevertheless, the public may not want the Gulf to deteriorate further. If risk of environmental degradation is a major concern, a different approach may be warranted. In such a situation, the risk concern may be met by modest reductions in N losses to the Gulf while carefully monitoring the Gulf on the one hand and learning more about the efficacy of N loss reduction efforts on the other (also known as adaptive management)[14].

The economic analysis gives guidance for crafting such a strategy. The analysis indicates that we can reduce N losses from agriculture in the 20% range through either nutrient restrictions or programs aimed at reducing N losses. We can also restore a modest number of acres of wetlands, 1 to 2 million, in a cost-effective manner. The strategies combined would not greatly affect farm prices or consumer food costs in comparison with other events in the past that have done so. However, some farms would face higher costs of following a program than others, and the specific incidence of this is unknown. For example, farms on marginal soils or in drier areas may have limited flexibility in trying to meet N-reduction goals. We also do not know the full cost of the programs themselves, be they wetlands restorations, N restrictions, or N loss reductions requiring incentives.

A moderate and mixed approach buys important benefits beyond the risk insurance that the problem is unlikely to get worse and may improve. We learn the costs of the programs and the associated benefits within the basin. We learn more about the timing delay of amelioration and the impacts of weather. We learn, over time, about the relationship between a given reduction in N losses and conditions in the Gulf. The critical component of this approach is monitoring. Continuous monitoring over a long period of time, something like 10 years, would be essential to learn what we need to know to determine better the efficacy and cost of reducing N losses further. If we are unwilling to make the commitment to monitoring and assessment, much of the benefit of understanding required for cost-effective approaches to reducing N losses will be missed. We should be able to reduce risk through decisions bounded by economic analysis of relative magnitudes and hold environmental degradation in check while we do so.

| | | |
|---|---|---|
| Received: | July | 18, 2001 |
| Revised: | September | 26, 2001 |
| Accepted: | October | 3, 2001 |
| Published: | October | 23, 2001 |
